# Supplementary material for: Multi-site integrated optical addressing of trapped ions
Source: Nat Commun. 2024 May 2;15:3709. doi: 10.1038/s41467-024-47882-5 (PMC11065861; doi:10.1038/s41467-024-47882-5)
Supplement: Supplementary file 1 — Supplementary Information [file 41467_2024_47882_MOESM1_ESM.pdf]

Supplementary Information:

Multi-site Integrated Optical Addressing of Trapped Ions

Joonhyuk Kwon[1,*], William J. Setzer[1], Michael Gehl[1], Nicholas Karl[1], Jay Van Der Wall[1], Ryan Law[1], Matthew G. Blain[1,2], Daniel Stick[1], Hayden J. McGuinness[1,†]

1. Sandia National Laboratories, Albuquerque, New Mexico 87185, USA;

2. Present Address: Quantinuum LLC, 303 S Technology Ct, Broomfield, CO 80021, USA;

∗ Corresponding author; e-mail: jookwon@sandia.gov

† Corresponding author; e-mail: hmcgui@sandia.gov

Supplementary Figures

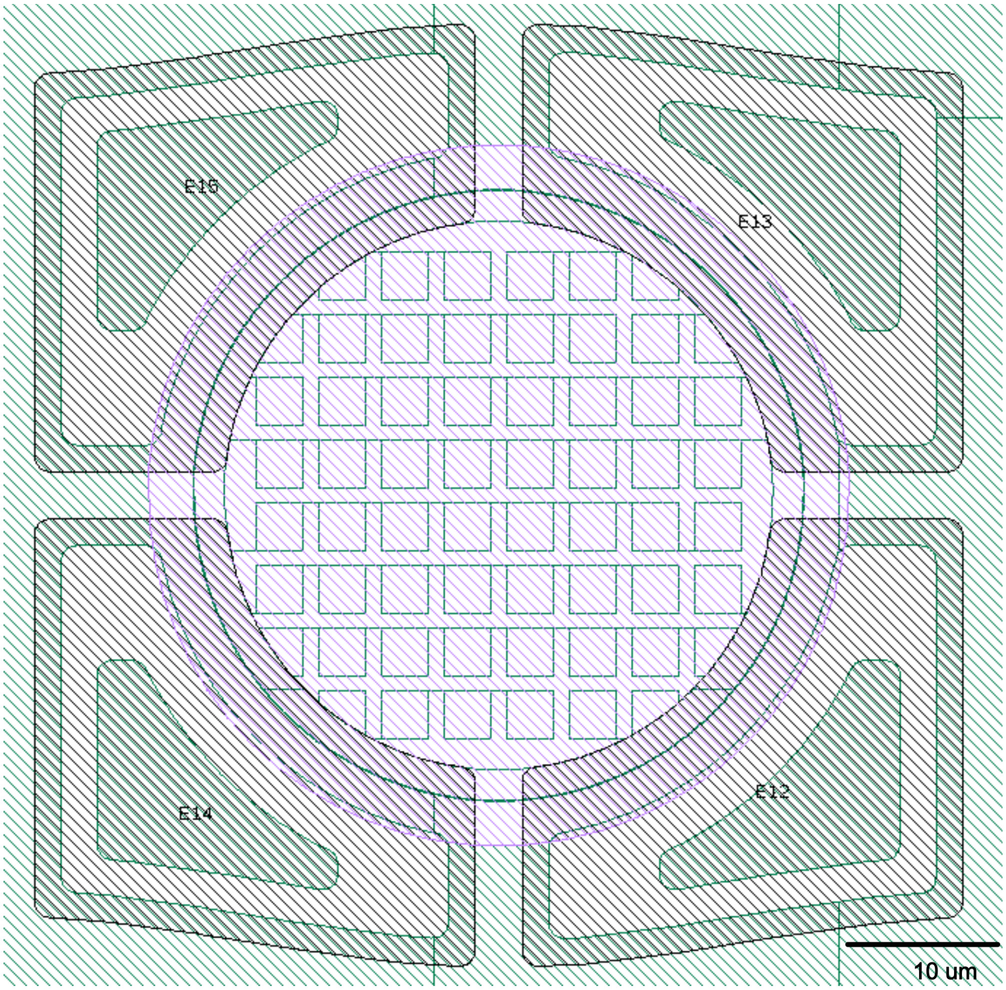

Supplementary Fig. 1. Lithography mask in the SPAD region A metal mesh is located on top of the SPAD (purple circle), consisting of $3\,\mu$m $\times$ $3\,\mu$m holes with $1\,\mu$m wide metal traces. The mesh and SPAD are surrounded by nearby electrodes, also shown in Fig. 1**a**.

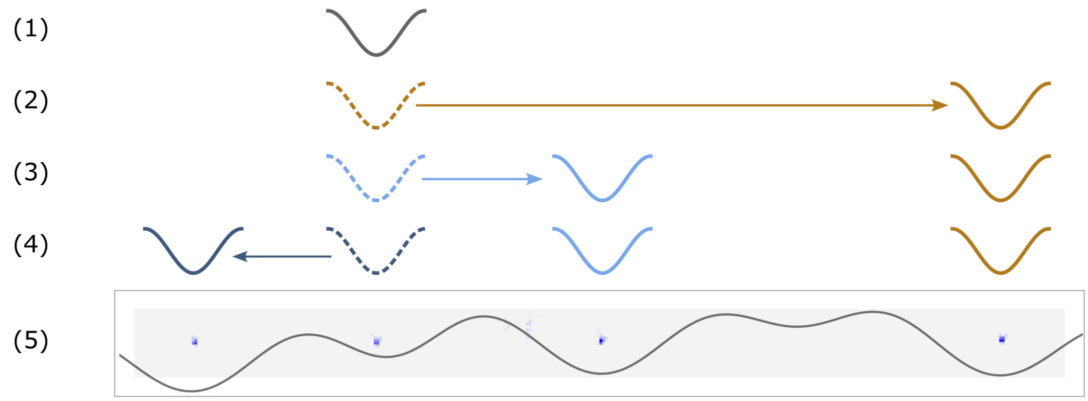

Supplementary Fig. 2. Multi-ion loading sequence Three waveguide-illuminated independent ions are loading sequentially, starting from one loading location that used free-space beams. 3+1 ions are trapped simultaneously by generating potential wells and loading/shuttling ions via steps (1) to (5). The potential plot at (5) represents a real four-ion solution that is used in this work, providing secular frequencies of $2\pi \times 1.02$MHz.

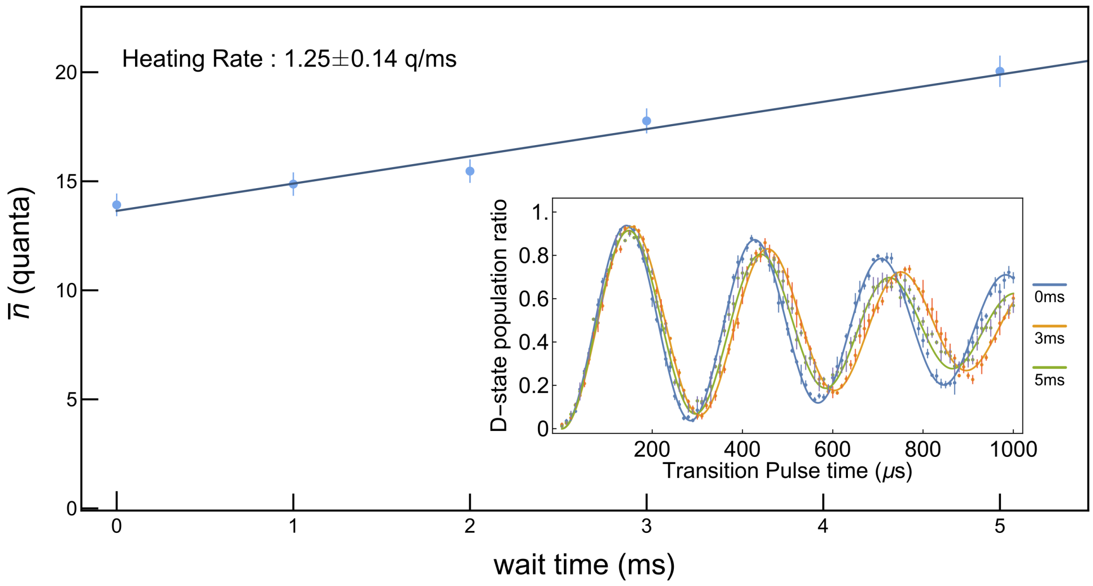

Supplementary Fig. 3. Heating rate measurement A heating rate of 1.25 ± 0.14 q/ms is measured using fully integrated beams for state preparation, cooling, and Rabi flopping, while a free-space beam is used for detection. **inset:** Measured Rabi flopping for different wait times.
